# A208 REAL WORLD EVIDENCE ANALYSIS WITH ADALIMUMAB BIOSIMILAR, MSB 11022, REPORTED WITH PATIENT CARE PROGRAM

**DOI:** 10.1093/jcag/gwac036.208

**Published:** 2023-03-07

**Authors:** S Cheung, P Heidari, D Truong

**Affiliations:** Fresenius Kabi Canada, Toronto, Canada

## Abstract

**Background:**

Injection site reactions (ISR), including injection site pain (ISP) are common adverse events reported with drugs administrated via subcutaneous injections. In clinical trials, the rates of ISR of adalimumab and adalimumab biosimilars range from 3% to 22.8% with citrate-buffer formulations, and from 1.7% to 16.6% with acetate-buffered and other formulations.

**Purpose:**

The objective of this study is to evaluate ISR of adalimumab biosimilar, MSB 11022, in real world setting.

**Method:**

Patients with immune mediated inflammatory diseases (IMIDs) enrolled with patient care program (PSP), and received at least one dose of adalimumab biosimilar, MSB 11022, were followed up. Incidences of injection site reactions, including injection site pain, burning sensation, bruise, erythema, hemorrhage, pruritus, and/or swelling were documented through unsolicited and solicited (outgoing following up calls with patients after their first injection through PSP contacts) reports. The data were collected between April 2021 and July 2022, and the rates of ISRs were assessed.

**Result(s):**

There were 2812 patients that met criteria through data collection period, with 219 cases of ISR. More than 99% of case reports were through solicited contacts, i.e., through case-manager follow up calls. Overall, rate of injection site reaction is 7.8% in patients with IMIDs treated with adalimumab biosimilar, MSB 11022. The rates of ISRs reported by disease are as follows: 6.8% of patients with Crohn's disease, 8.7% with ulcerative colitis, 8.8% with rheumatoid arthritis, 6.5% with psoriatic arthritis, 7.8% ankylosing spondylitis, 6.1% uveitis, 8.3% with hidradenitis suppurativa, and 11.1% of juvenile rheumatoid arthritis.

**Conclusion(s):**

Real world data provided evidence that ISR rate of adalimumab biosimilar, MSB 11022, is within the ranges of clinical trial data of adalimumab with citrate-buffer, and other formulations. As far as we know, this is the first real world evidence reported for ISR among adalimumab biosimilars with IMID patients.

**Please acknowledge all funding agencies by checking the applicable boxes below:**

None

**Disclosure of Interest:**

None Declared

